# Systematic Review of Tissue-Engineered Vascular Grafts

**DOI:** 10.3389/fbioe.2021.771400

**Published:** 2021-11-03

**Authors:** David Durán-Rey, Verónica Crisóstomo, Juan A. Sánchez-Margallo, Francisco M. Sánchez-Margallo

**Affiliations:** ^1^ Laparoscopy Unit, Jesús Usón Minimally Invasive Surgery Centre, Cáceres, Spain; ^2^ Cardiovascular Unit, Jesús Usón Minimally Invasive Surgery Centre, Cáceres, Spain; ^3^ Centro de Investigacion Biomédica en Red de Enfermedades Cardiovasculares (CIBERCV), Instituto de Salud Carlos III, Madrid, Spain; ^4^ Bioengineering and Health Technologies Unit, Jesús Usón Minimally Invasive Surgery Centre, Cáceres, Spain; ^5^ Scientific Direction, Jesús Usón Minimally Invasive Surgery Centre, Cáceres, Spain

**Keywords:** tissue-engineered vascular graft, patency, thrombosis, scaffold, animal model, human patient

## Abstract

Pathologies related to the cardiovascular system are the leading causes of death worldwide. One of the main treatments is conventional surgery with autologous transplants. Although donor grafts are often unavailable, tissue-engineered vascular grafts (TEVGs) show promise for clinical treatments. A systematic review of the recent scientific literature was performed using PubMed (Medline) and Web of Science databases to provide an overview of the state-of-the-art in TEVG development. The use of TEVG in human patients remains quite restricted owing to the presence of vascular stenosis, existence of thrombi, and poor graft patency. A total of 92 original articles involving human patients and animal models were analyzed. A meta-analysis of the influence of the vascular graft diameter on the occurrence of thrombosis and graft patency was performed for the different models analyzed. Although there is no ideal animal model for TEVG research, the murine model is the most extensively used. Hybrid grafting, electrospinning, and cell seeding are currently the most promising technologies. The results showed that there is a tendency for thrombosis and non-patency in small-diameter grafts. TEVGs are under constant development, and research is oriented towards the search for safe devices.

## 1 Introduction

Cardiovascular diseases (CVDs) are the main cause of death globally (World Health Organization, 2020). The narrowing or blockage of blood vessels are disorders that induce reduced blood flow and tissue damage due to poor nutrient provision (Pashneh-Tala et al., 2015). Annual mortality from CVDs is expected to increase to 23.3 million people worldwide by 2030 (Mathers and Loncar, 2006).

A change in lifestyle, including a healthy and balanced diet, could be adequate to prevent CVD. However, surgical and pharmaceutical intervention are often required (Abdulhannan et al., 2012). Endovascular surgeries, including angioplasty, can be used to mitigate these diseases (Pashneh-Tala et al., 2015). However, conventional surgeries using autologous saphenous veins, radial arteries, or internal mammary artery transplants—which create a bypass to restore normal blood flow—are often required (Row et al., 2017). Many studies have been conducted aiming to treat vascular disorders through the use of vascular grafts, and their efficacy in animal models and human patients has been demonstrated.

In certain patients, and especially in the elderly, the use of autologous grafts may not be possible (Mozaffarian et al., 2015). Consequently, new technologies, such as tissue engineering, have begun to be developed. Tissue-engineered vascular grafts (TEVGs) show promise as a clinical treatment (Radke et al., 2018).

Synthetic materials have been studied for the creation of vascular grafts for more than 50 years. In the middle of the last century, vascular grafts were developed using two types of synthetic materials: polytetrafluoroethylene (PTFE, Teflon^®^) and polyethylene terephthalate (PET, Dacron^®^). Promising results have been obtained in aorto-iliac replacements and in arteries with medium-sized diameters (6–8 mm). However, these materials have not produced satisfactory results in small-caliber grafts owing to thrombus formation or poor patency rates (Lovett et al., 2007; Pashneh-Tala et al., 2015).

Weinberg and Bell created the first TEVG designed with biosynthetic materials in 1986, created from collagen gel tubes and cultures of vascular cells, such as bovine aortic endothelial cells (EC), smooth muscle cells (SMCs), and fibroblasts from adventitia (Weinberg and Bell, 1986). In the following years, other authors conducted studies using similar materials (Pashneh-Tala et al., 2015). However, one of the problems encountered was that the vascular grafts did not support arterial pressure. Conversely, the use of nondegradable materials may have harmful effects in organisms (L’Heureux et al., 2007). Different vascular graft scaffolds have been developed to correct these effects, wherein animal cells were seeded in partially resorbable polymers (Shum-Tim et al., 1999; Shin’oka et al., 2001). The developed animal models were able to reproduce the different mechanical vascular properties and yielded satisfactory results. However, when human cells were used, vascular grafts did not exhibit adequate mechanical properties for potential applications in humans (Poh et al., 2005).

Ideal TEVGs should be designed with scaffolds that shape the graft with an adhesive matrix mainly consisting of fibrin and vascular cells (Baguneid et al., 2006). In addition, TEVGs should have high strength to prevent rupture and facilitate manipulation, a design capable of replacing the host’s tissue, and an adequate graft size for clinical use (Mahara et al., 2015).

Many of the challenges that hampered the development of vascular grafts have been overcome, particularly during the last decade. Nevertheless, there are still many aspects to be improved and problems to be solved to guarantee their safe use. A wide variety of new materials and technologies have been developed to create TEVGs, each providing different properties to these grafts. Therefore, we believe that this systematic review provides an update of the results recently published in the scientific literature, emphasizing the methods, and innovations used in the development of TEVGs. It also summarizes the current results obtained in animal models and human patients and influence of the graft diameter and heparin use on the occurrence of thrombosis and patency in these grafts. Thus, the objective of this work is to provide an overview of the current state-of-the-art in the development of TEVGs to demonstrate the advances and challenges faced over the last decade and present the objectives and clinical applications pursued by the authors.

## 2 Materials and Methods

### 2.1 Search Strategy

A structured bibliographical search was conducted in the PubMed and Web of Science databases. We used a set of keywords related to TEVGs to identify relevant studies published from September 16, 2010, to September 15, 2020 (see Supplementary Materials, full search strategy). This systematic review has been registered on PROSPERO with the registration number CRD42020191561.

### 2.2 Selection of Articles

A series of inclusion and exclusion criteria were considered to select the articles that best applied for our objectives (see Supplementary Materials). In general, articles whose subject was TEVG, written in English, and including surgical procedures and follow-up in animal models or human patients were selected. A flowchart showing the different phases of the systematic review was designed according to the Preferred Reporting Items for Systematic Reviews and Meta-Analysis (PRISMA) statement for reporting systematic reviews and meta-analyses (Moher et al., 2015).

### 2.3 Statistical Analysis

A meta-analysis of the influence of the vascular graft diameter on thrombosis occurrence and patency was performed for the different models analyzed. For this purpose, the normality of the data distribution was analyzed using the Kolmogorov–Smirnov test. As the distribution of the data was not normal, we used the nonparametric Mann–Whitney U-test for independent samples to compare the data. Additionally, the relationship between qualitative variables of heparin use during surgery and graft patency and the onset of thrombosis was analyzed. The Fisher’s exact test was used for this purpose. All statistical analyses were conducted using R (version 4.0.0, R Foundation for Statistical Computing, Vienna, Austria). For all tests, *p* < 0.05 was considered to be statistically significant. Only the models with adequate data for statistical analysis were shown in the graphs.

## 3 Results

### 3.1 Selection of Articles

**FIGURE 1:**
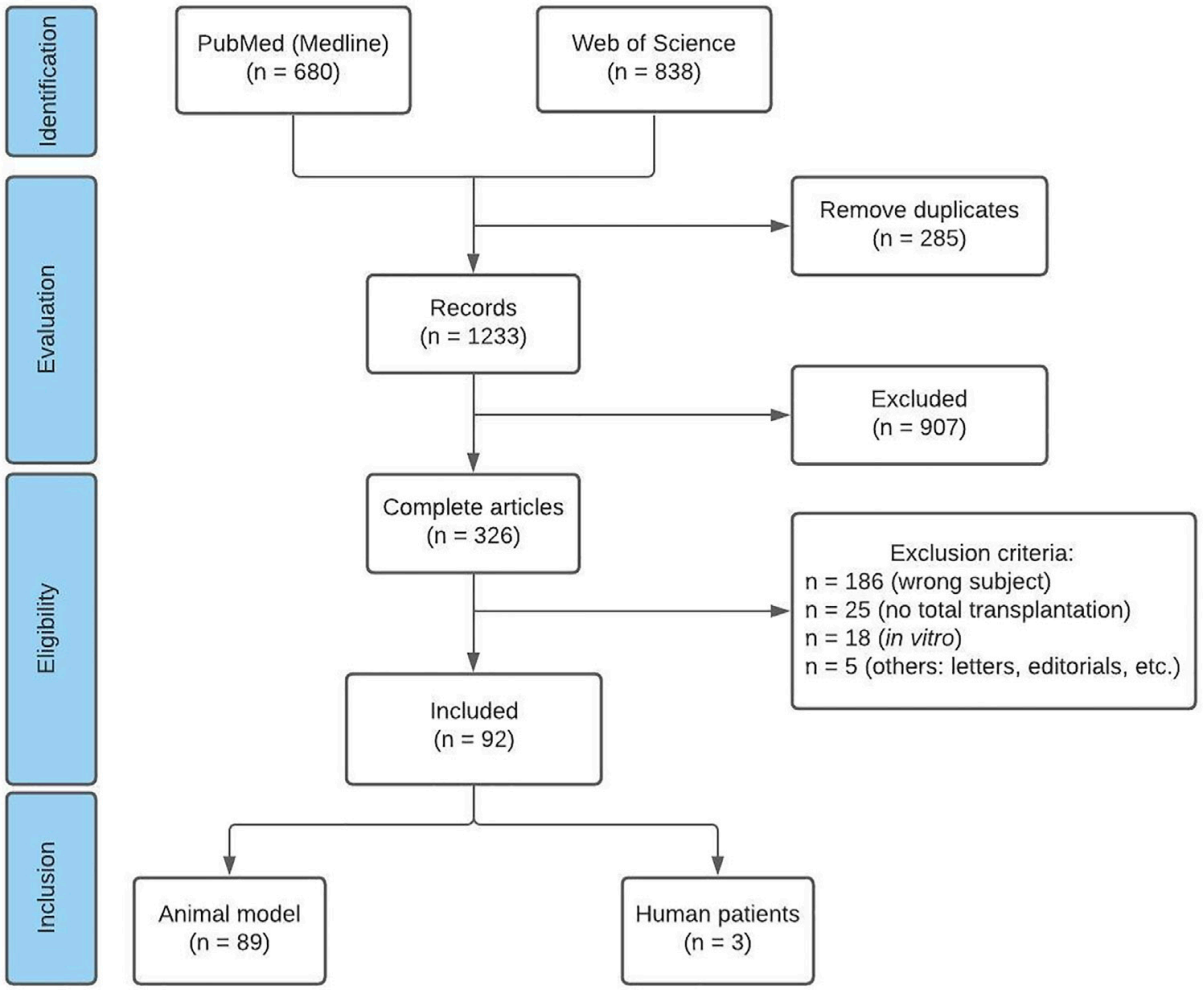
PRISMA flow diagram. Diagram showing the manuscript selection process in accordance with PRISMA guidelines.

A total of 680 and 838 records were identified in PubMed and Web of Science databases, respectively. Based on the analysis of the title and abstract of each article, 907 records were excluded because: 1) the subject did not comply with the goal of this review (431); 2) they were types of studies that did not meet the inclusion criteria (454); or 3) they were in a language other than English (22). After completely reading these articles, 92 studies were selected, 89 of which used animal models and three were conducted with human patients (Figure 1).

### 3.2 Animal Models and Human Trials for TEVG

Regarding experimental animal models, studies with rodents, ovine, pigs, rabbits, dogs, and baboons were included in this systematic review. In addition, human trials were also analyzed in the review (Table 1).

**TABLE 1:** Studies with animal models and human patients included in the systematic review.

Animal model/human trials	Number of studies	Percentage of total (%)	References
Rodents	44	46.31	Lovett et al. (2010), Song et al. (2010), Harrington et al. (2011), Hibino et al. (2011), Hwang et al. (2011), Assmann et al. (2012), Quint et al. (2012), De Valence et al. (2013), Conconi et al. (2014), Lee et al. (2014), Tara et al. (2014), Udelsman et al. (2014), Itoh et al. (2015), Tara et al. (2015), Wu et al. (2015), Fukunishi et al. (2016), Gui et al. (2016), Hibino et al. (2016), Jiang et al. (2016), Khosravi et al. (2016), Tondreau et al. (2016), Wang et al. (2016), Yang et al. (2016), Li et al. (2017), Maxfield et al. (2017), Negishi et al. (2017), Best et al. (2018), Lee et al. (2018), Li et al. (2018), Qin et al. (2018), Shafiq et al. (2018), Wu et al. (2018), Xu et al. (2018), Aubin et al. (2019), Bai et al. (2019), Best et al. (2019), Katsimpoulas et al. (2019), Liu et al. (2019), Ran et al. (2019), Shi et al. (2019), Smith et al. (2019), Sologashvili et al. (2019), Yamanami et al. (2019)
Ovine	19	20	Cummings et al. (2012), Koenneker et al. (2012), Tillman et al. (2012), Meier et al. (2014), Stacy et al. (2014), Row et al. (2015), Aper et al. (2016), Fukunishi et al. (2016), Koobatian et al. (2016), Syedain et al. (2016), Fukunishi et al. (2017), Ju et al. (2017), Pepper et al. (2017), Ong et al. (2017a), Madhavan et al. (2018), Valencia-Rivero et al. (2018), Weber et al. (2018), Alessandrino et al. (2019), Wolf et al. (2019)
Pigs	13	13.68	Quint et al. (2011), Mrówczyński et al. (2014), Koens et al. (2015), Mahara et al. (2015), Pellegata et al. (2015), Rothuizen et al. (2016), Dahan et al. (2017), Iacobazzi et al. (2018), Valencia-Rivero et al. (2018), Alessandrino et al. (2019), Itoh et al. (2019), Wang et al. (2019), Yeung et al. (2019)
Rabbits	7	7.37	Zhao et al. (2012), McIlhenny et al. (2015), Hu et al. (2017), Tseng et al. (2017), Jin et al. (2019a), Jin et al. (2019b), Kajbafzadeh et al. (2019)
Dogs	7	7.37	Matsumura et al. (2012), Aytemiz et al. (2013), Matsumura et al. (2013), Isayama et al. (2014), Ma et al. (2017), Furukoshi et al. (2019), Jang et al. (2020)
Human patients	3	3.16	Sugiura et al. (2018), Herrmann et al. (2019), Kirkton et al. (2019)
Baboons	2	2.11	Prichard et al. (2011), Syedain et al. (2017)

### 3.3 Place of Implantation

Grafts were implanted in the abdominal aorta, carotid, pulmonary, femoral, and iliac arteries, and the portal, cava, and jugular veins. There were some studies where bypass or shunt procedures were performed (Prichard et al., 2011; Koenneker et al., 2012; Tillman et al., 2012; Syedain et al., 2017; Ong et al., 2017a; Valencia-Rivero et al., 2018; Bai et al., 2019; Furukoshi et al., 2019; Itoh et al., 2019).

### 3.4 Design of TEVG

Four groups were differentiated in this systematic review according to the materials used in the graft design: 1) TEVG created with biodegradable polymers, 2) TEVG developed with natural materials, 3) TEVG created with biodegradable polymers and natural materials or hybrid grafts, and 4) tissue-engineered scaffolds or acellular tissue-engineered grafts (Table 2).

**TABLE 2:** Materials used in the vascular graft design.

Materials	Number of studies	Percentage of total (%)	References
Biodegradable polymers	11	11.96	Cummings et al. (2012), Harrington et al. (2011), Hibino et al. (2011), Lee et al. (2014), Stacy et al. (2014), Udelsman et al. (2014), Gui et al. (2016), Pepper et al. (2017), Sugiura et al. (2018), Jang et al. (2020), Luo et al. (2020)
Natural materials	17	18.48	Quint et al. (2011), Koenneker et al. (2012), Tillman et al. (2012), Zhao et al. (2012), Meier et al. (2014), Itoh et al. (2015), McIlhenny et al. (2015), Pellegata et al. (2015), Row et al. (2015), Wu et al. (2015), Aper et al. (2016), Rothuizen et al. (2016), Dahan et al. (2017), Ma et al. (2017), Tseng et al. (2017), Iacobazzi et al. (2018), Herrmann et al. (2019), Itoh et al. (2019)
Hybrid grafts	5	5.43	Hu et al. (2017), Ju et al. (2017), Madhavan et al. (2018), Shafiq et al. (2018)
Tissue-engineered scaffolds	59	64.13	Lovett et al. (2010), Song et al. (2010), Hwang et al. (2011), Prichard et al. (2011), Assmann et al. (2012), Matsumura et al. (2012), Quint et al. (2012), Aytemiz et al. (2013), De Valence et al. (2013), Matsumura et al. (2013), Conconi et al. (2014), Isayama et al. (2014), Mrówczyński et al. (2014), Tara et al. (2014), Koens et al. (2015), Mahara et al. (2015), Tara et al. (2015), Fukunishi et al. (2016), Hibino et al. (2016), Jiang et al. (2016), Khosravi et al. (2016), Koobatian et al. (2016), Syedain et al. (2016), Tondreau et al. (2016), Wang et al. (2016), Yang et al. (2016), Fukunishi et al. (2017), Li et al. (2017), Maxfield et al. (2017), Negishi et al. (2017), Ong et al. (2017a), Syedain et al. (2017), Best et al. (2018), Lee et al. (2018), Li et al. (2018), Qin et al. (2018), Valencia-Rivero et al. (2018), Weber et al. (2018), Wu et al. (2018), Xu et al. (2018), Alessandrino et al. (2019), Aubin et al. (2019), Bai et al. (2019), Best et al. (2019), Furukoshi et al. (2019), Jin et al. (2019a), Jin et al. (2019b), Kajbafzadeh et al. (2019), Katsimpoulas et al. (2019), Kirkton et al. (2019), Liu et al. (2019), Ran et al. (2019), Shi et al. (2019), Smith et al. (2019), Sologashvili et al. (2019), Wang et al. (2019), Wolf et al. (2019), Yamanami et al. (2019), Yeung et al. (2019)

### 3.5 Statistical Results

**FIGURE 2:**
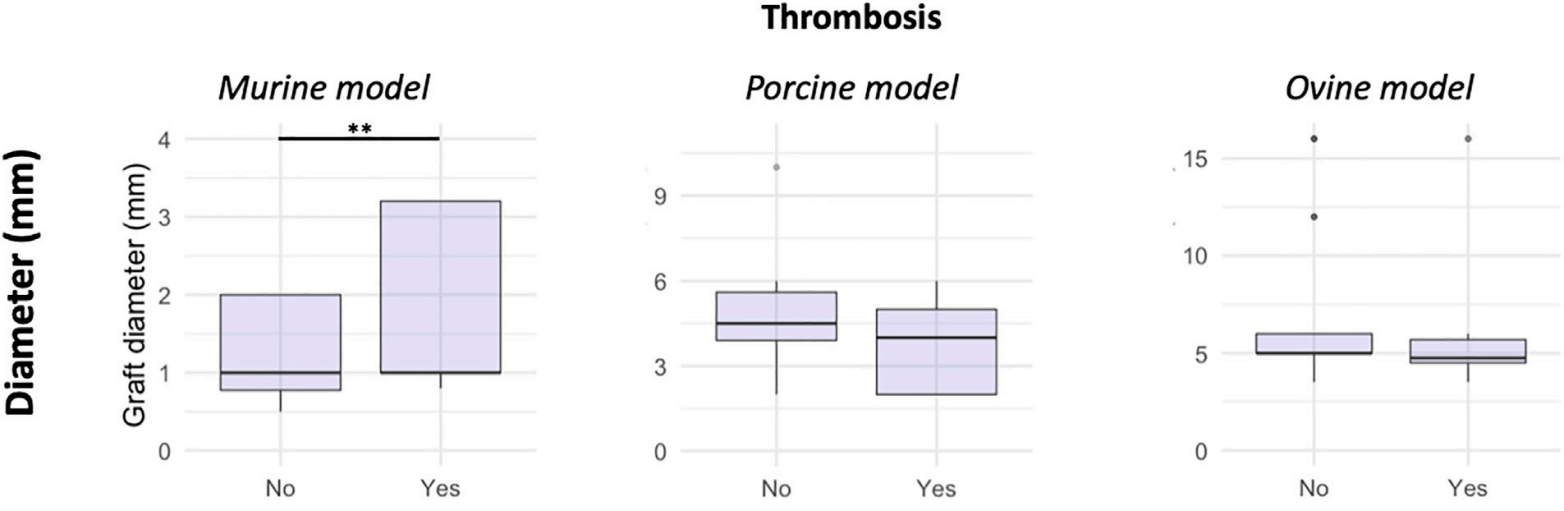
Box plots representing the relationship between the graft diameter and onset of thrombosis. The boxed represent the interquartile (25th–75th percentiles) range; the horizontal line within each box represents the median; and the whiskers represent the range (**p < 0.01).

**FIGURE 3:**
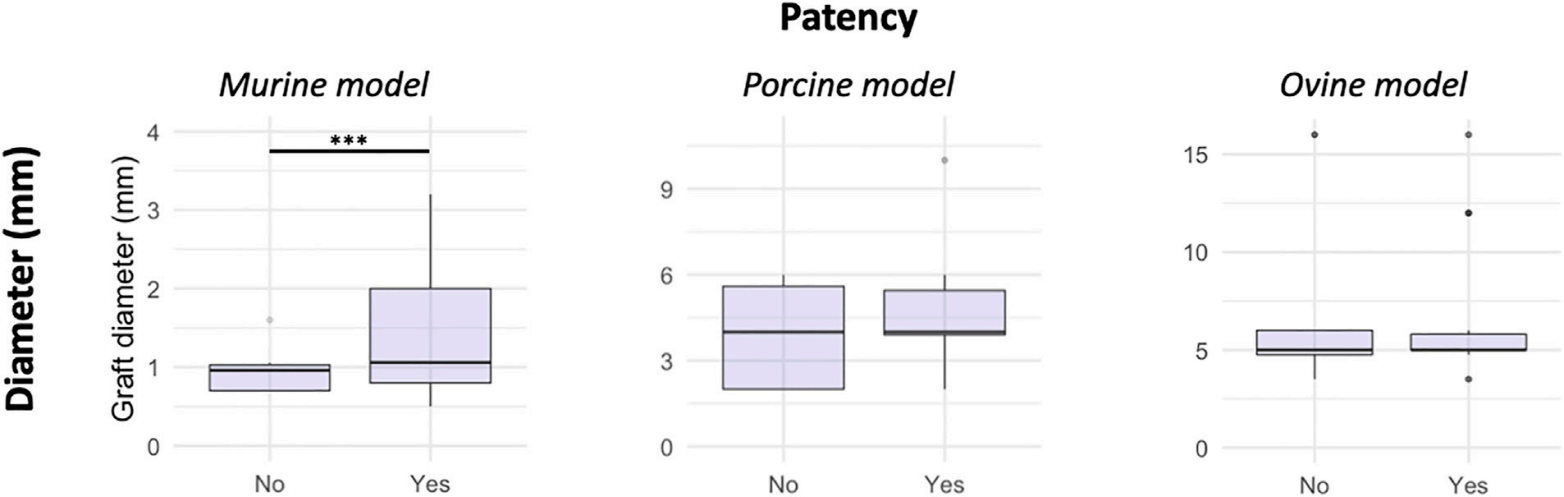
Box plots representing the relationship between the graft diameter and its patency. The boxes represent the interquartile (25th–75th percentiles) range; the horizontal line within each box represents the median; and the whiskers represent the range (***p < 0.001).

Rodents led to a greater occurrence of thrombosis for large-diameter grafts (Figure 2). Conversely, grafts with a large diameter in rodents had good patency (Figure 3). Both porcine and ovine models did not show statistically significant differences in the relationship between the graft diameter and onset of thrombosis (Figure 2) and in the relationship between the graft diameter and patency (Figure 3).

**FIGURE 4:**
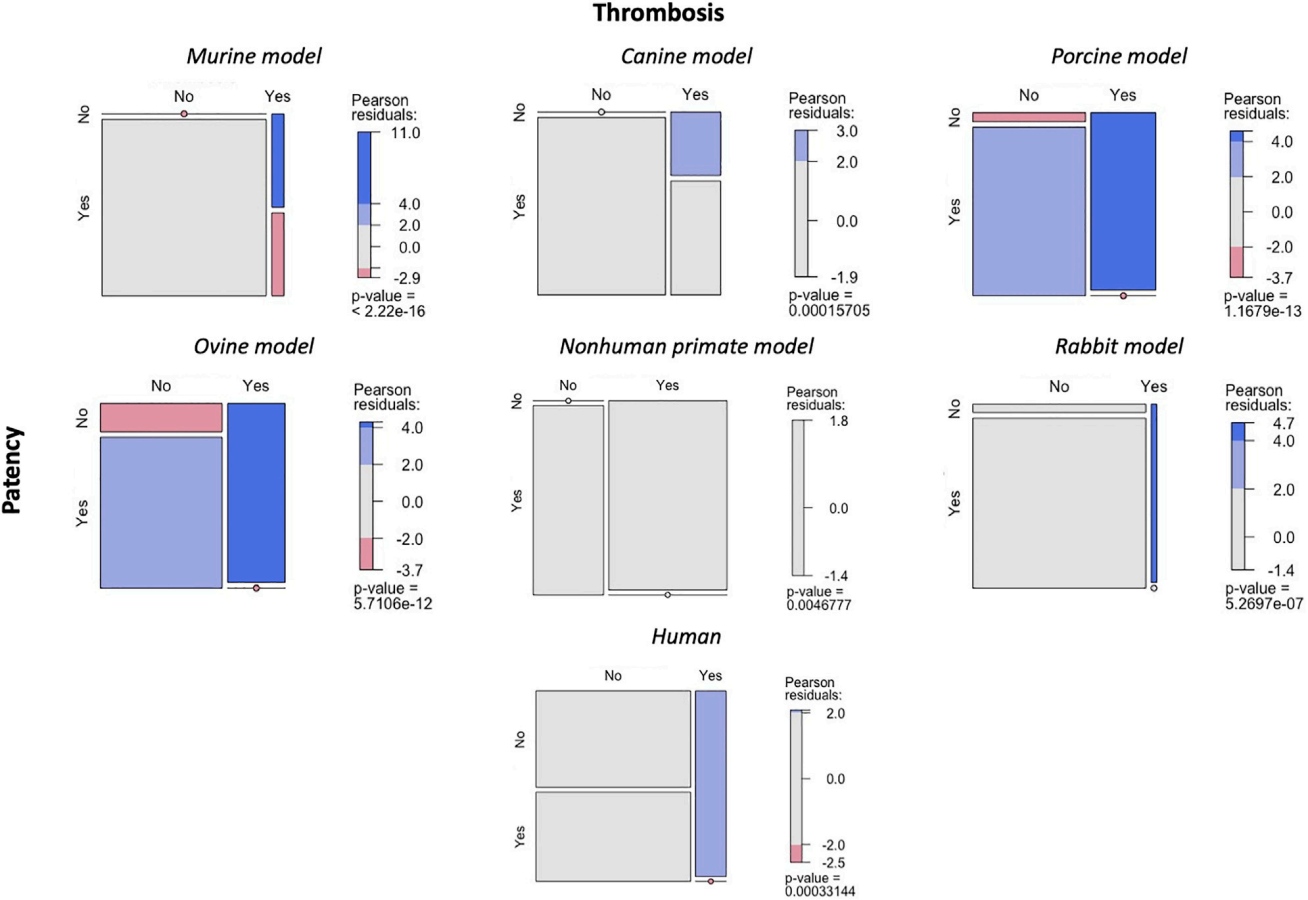
Relationship between graft patency and occurrence of thrombosis. Mosaic plot used to evaluate the association between the graft patency and onset of thrombosis.

There was a statistically significant correlation between the patency of the vascular graft and the absence of thrombus in murine, ovine, porcine, rabbit, and canine models (Figure 4). There was a correlation between the non-patency of the vascular graft and the onset of thrombosis during surgery in the nonhuman primate model. In the case of human patients, there was a correlation between the nonpatency of the grafts and the occurrences of thrombi.

**FIGURE 5:**
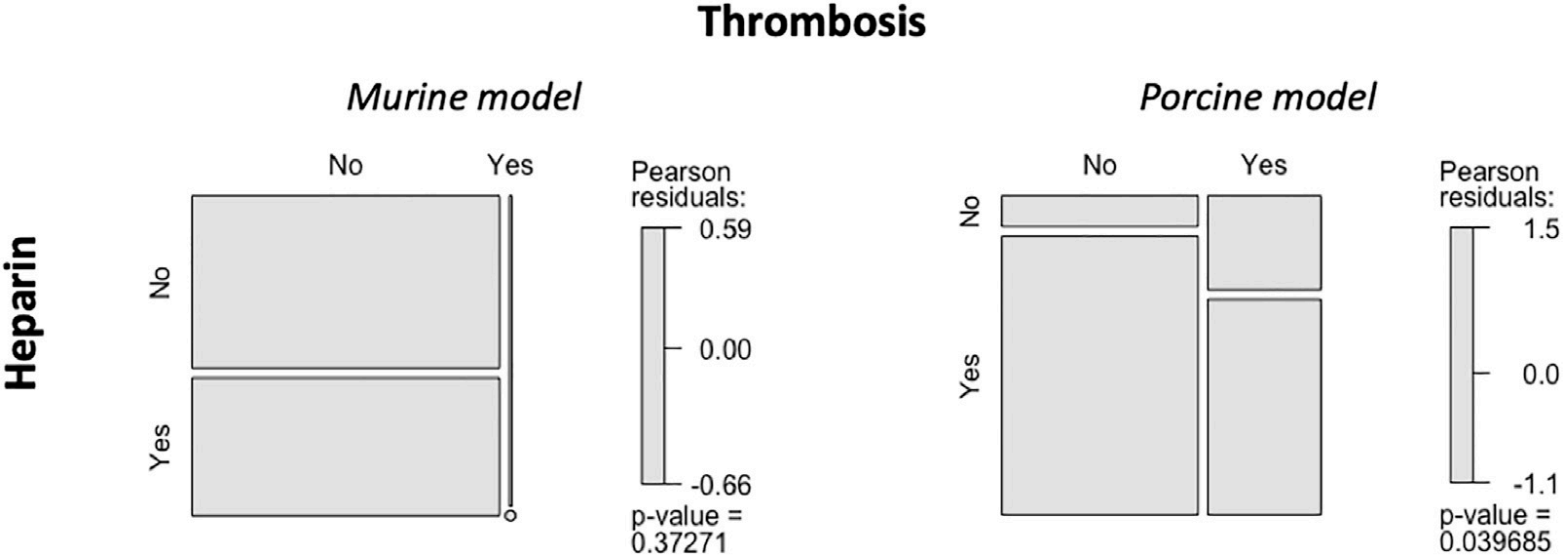
Relationship between the use of heparin and occurrence of thrombosis. Mosaic plot used to evaluate the association between the use of heparin and onset of thrombosis.

There was no relationship between the use of heparin and the onset of thrombosis during surgery in the murine and porcine models (Figure 5).

## 4 Discussion

Conventional surgery with the use of vascular grafts, such as autologous transplants, is a treatment for many CVDs (e.g., atherosclerosis) (Row et al., 2017). However, such autologous vascular grafts are often unavailable (Mozaffarian et al., 2015). Therefore, the development of tissue-engineered vascular grafts (TEVGs) represents an innovative area of research that aims to offer continuous availability. Tissue engineering is a multidisciplinary field combining biomedical engineering, material science, regenerative medicine, and immunology (Best et al., 2018). Although many studies using various materials and methods for the development of TEVGs have been published (Row et al., 2017), some of the defects they entail, such as the occurrences of thrombi, have not been fully corrected yet. In this work, we conducted a comprehensive review of recently published results in the field of TEVG development, both in animal models and for use in humans. Emphasis has been placed on the influence of the graft diameter and the use of heparin on the occurrence of thrombi and patency of the graft.

### 4.1 Animal Models for TEVG

Six experimental animal models have been included in this review. The ideal animal model should exhibit a cardiovascular anatomy and physiology very similar to that of humans. However, each animal model has a series of peculiarities (Table 3). Rodents were the most commonly used model in the studied literature owing to their low cost. Further, besides being ideal for biocompatibility and cell infiltration studies (Pashneh-Tala et al., 2015), they provide information about the molecular and cellular bases of cardiovascular biology (Lelovas et al., 2014). Furthermore, there are genetically modified strains and immune-deficient rodents that are used to simulate cardiovascular disease in humans (Bergmeister et al., 2019). Nevertheless, cardiovascular physiology, thrombogenicity, and hemostasis mechanisms are different from those in humans (Pashneh-Tala et al., 2015; Bergmeister et al., 2019). It seems that the biocompatibility of rodents allows them to withstand TEVG without the use of heparin to prevent the appearance of thrombosis and to have a good long-term patency. Nevertheless, the use of heparin is advisable to prevent possible thrombus formation (Aslani et al., 2020). The second most used animal model was the ovine model. Unlike rodents, sheep have thrombogenicity mechanisms and fibrinolysis system very similar to humans, as well as endothelialization and neointimal formation of blood vessels (Pashneh-Tala et al., 2015; Bergmeister et al., 2019). However, they have a high tendency of hypercoagulability (Pashneh-Tala et al., 2015). Porcine have a cardiovascular anatomy very similar to that of humans; therefore, there are numerous cardiovascular studies using porcine models in the scientific literature (Lelovas et al., 2014). However, because porcine grow rapidly, they become difficult to handle; thus, some studies have utilized miniature pigs (Mahara et al., 2015; Alessandrino et al., 2019; Itoh et al., 2019; Wang et al., 2019). Extensive heparin has been used in porcine models but there was no evidence for the formation of thrombi during surgery. The immune responses against vascular grafts were very intense and tended to hypercoagulate after surgical intervention (Mulier et al., 2012; Pashneh-Tala et al., 2015). Thus, the use of heparin was fundamental in this animal model. Further, muscular spams are very common in the arteries of pigs and are fragile (Bergmeister et al., 2019). Rabbits present thrombogenicity pathways comparable to other models (Pashneh-Tala et al., 2015), and hemostasis and endothelialization are more similar to that of humans than in the case of rodents. There are some heritable disease models that can be used for certain pathologies, such as hypercholesteremic Watanabe rabbit (Bergmeister et al., 2019). Nevertheless, their vascular physiology is quite different from human physiology (Pashneh-Tala et al., 2015). In the case of the canine model, the main advantages are that the anatomy has been extensively studied and lack of spontaneous endothelialization, which makes their control and monitoring advantageous. However, thrombogenicity presents different mechanisms with respect to the human model (Pashneh-Tala et al., 2015), and have a potent fibrinolytic system (Bergmeister et al., 2019). There are ethical aspects related to a diminished acceptance from an experimental point-of-view. Finally, two studies were conducted with nonhuman primates (Prichard et al., 2011; Syedain et al., 2017). This model has clear similarities with humans in terms of physiology, cardiovascular anatomy, and thrombogenicity mechanisms. However, this model is costly and presents ethical considerations regarding its use (Pashneh-Tala et al., 2015).

**TABLE 3:** Summary of the advantages and disadvantages of animal models analyzed.

Animal model	Advantages	Disadvantages
Rodents	Low cost	Cardiovascular physiology
	Biocompatibility and cell infiltration studies	Thrombogenicity mechanisms
	Genetically modified strains	Hemostasis mechanisms
Ovine	Thrombogenicity mechanisms	Hypercoagulability
	Fibrinolysis system	
	Endothelialization and neointimal formation	
Porcine	Cardiovascular anatomy	Grow rapidly
	Translational studies	Hypercoagulability
Rabbits	Thrombogenicity mechanisms	Vascular physiology
	Heritable disease models	
Canine	Anatomy extensively studied	Thrombogenicity mechanisms
	Lack of spontaneous endothelialization	Potent fibrinolytic system
		Ethical aspects
Nonhuman primates	Physiology	High cost
	Cardiovascular anatomy	Ethical considerations
	Thrombogenicity mechanisms	

According to the results found, the ovine and porcine models seem to be appropriate models for TEVGs research. On the one hand, ovine model has an anatomy and hemodynamic mechanism quite similar to human. Further, these animals have a long neck, greatly facilitates surgery. Moreover, porcine is widely used for translational studies due to its strong physiological resemblance to humans. All studies related to new TEVGs should evaluate their biocompatibility, patency, hemodynamic factors, and cell infiltration, among others. As each animal model has a series of advantages and limitations, it is important to know the different characteristics of each animal model to allow their results and possible effects in human patients to be interpreted correctly.

### 4.2 Human Trials with TEVG

Regarding human patients, this systematic review found three studies conducted in human published in 2018 (Sugiura et al., 2018) and 2019 (Herrmann et al., 2019; Kirkton et al., 2019). This is indicative of the fact that considerable progress has been achieved in the field of TEVGs. However, the use of these vascular grafts in humans remains very restricted owing to certain limitations, such as the presence of stenosis, existence of thrombi, and poor graft patency, among others. Kirkton et al. (2019) developed a vascular graft composed of primary human vascular cells isolated from cadaveric donors and seeded on polyglycolic acid (PGA) scaffolds. Scaffolds were decellularized to remove cellular antigens and were recellularized by vascular cells and noninflammatory host progenitors. Arteriovenous grafts with an internal diameter of 6 mm were implanted in the upper arm in 60 patients with end-stage renal disease to provide access for hemodialysis. The mean follow-up period was 3.8 years. Thromboses or pseudoaneurysms owing to cannulation trauma occurred in 13/60 patients. Herrmann et al. (2019) presented a vascular graft design composed of saphenous veins harvested from multiple organ donors that were cryopreserved, de-endothelialized, and seeded with human, autologous, venous EC from patient vein segments. Fifteen vascular grafts with diameters in the range of 3–6 mm were implanted by coronary artery bypass in 12 patients. The mean survival of patients after surgery was 9.1 ± 1.8 years. At the 6-months follow-up, the vascular graft patency was 80%. It decreased to 50% at 9 months, and at the endpoint, 7/12 patients (8/15 grafts) had graft occlusions or stenosis. Finally, Sugiura et al. (2018) implanted vascular grafts that consisted of woven poly-L-lactide acid (PLLA) or PGA coated with a copolymer sealant solution of poly-L-lactic-co-ε-caprolactone (PLCL) in 25 children. The grafts were seeded with autologous bone marrow mononuclear cells (BM-MNCs). The graft size ranged from 12 to 24 mm. These grafts were implanted as extracardiac total cavopulmonary connections. The mean follow-up period was 11.1 years. There were no lethal complications related to the graft, but a thrombus was detected in one patient, and seven other patients presented asymptomatic stenosis. Asymptomatic complications were diagnosed in the human studies mentioned above (Sugiura et al., 2018; Herrmann et al., 2019; Kirkton et al., 2019). Long-term research is needed to study all the side effects that these TEVGs could produce, thus achieving safe and satisfactory use. The use of TEVGs is still not completely safe in clinical studies due to the high failure rate, such as presence of stenosis, thrombi, or other complications.

### 4.3 Materials Used in the Design of TEVGs

TEVGs should resemble native blood vessels as closely as possible and have the ability to remodel, grow, self-repair, and respond to the immediate environment (Chlupác et al., 2009). Each article analyzed in this review created its own TEVG, although there were certain materials that were commonly used, such as biodegradable polymers, or biological materials. Notably, some researchers used heparin in the design of TEVGs (Jiang et al., 2016; Koobatian et al., 2016; Hu et al., 2017; Ju et al., 2017; Madhavan et al., 2018; Xu et al., 2018; Jin et al., 2019a; Jin et al., 2019b; Ran et al., 2019; Shi et al., 2019; Smith et al., 2019; Wang et al., 2019). The addition of heparin to materials prevents thrombosis and enhances biocompatibility (Aslani et al., 2020).

The most commonly studied and used polymer is PGA, which has high level of flexibility and lacks of inflammatory response (Pepper et al., 2017; Sugiura et al., 2018; Luo et al., 2020). When seeded with human EC in vascular grafts, PGA can withstand mechanical stress equivalent to the aortic pressure (Chlupác et al., 2009; Mauri et al., 2013). However, PGA should be used in association with other polymers due to short degradation time (6–8 weeks), which is too fast for vascular clinical applications (Leal et al., 2021). On the other hand, PLCL is a copolymer of lactic acid and caprolactone, which presents good biocompatibility and slow degradation (Hu et al., 2017; Pepper et al., 2017). Authors used the combination of PGA with PLCL to increase graft degradation time (Hibino et al., 2011; Lee et al., 2014; Stacy et al., 2014; Hibino et al., 2016; Pepper et al., 2017; Sugiura et al., 2018). Polylactic acid (PLA) is a polymer with a very similar structure and mechanical properties to PGA, but with a longer degradation time (Naito et al., 2011; Ong et al., 2017b). However, PLA exhibits a hydrophobic structure, which interferes in cell adhesion and proliferation (Leal et al., 2021). PLLA is an isomeric form of PLA and is the most studied polymer for cardiovascular tissue engineering applications (Mishra, 2015; Ong et al., 2017b), and the studies showed improved cell viability (Sugiura et al., 2018). Polycaprolactone (PCL) presents mechanical properties that exceed those of natural vessels, such as maximum stress or tensile strength. In addition, PCL has a good biocompatibility and slow biodegradability (Fukunishi et al., 2016; Shafiq et al., 2018). Nevertheless, PCL has hydrophobic characteristics, so needs to be combined with other polymers.

Contrarily, there were TEVGs created from biological materials. Collagen has low antigenicity, high biocompatibility, and enhances cell adhesion and proliferation (Shafiq et al., 2018; Itoh et al., 2019). Further, this material is the main component of the extracellular matrix (ECM) (Ong et al., 2017b). Chitosan is a new material use in TEVG, and provides low toxicity and anticoagulant properties, as well as inhibiting inflammation, and modifying viability chemically (Wu et al., 2015; Fukunishi et al., 2016). Elastin is part of ECM and maintains the elasticity of the blood vessels under blood pressure (Hu et al., 2017). The use of this material prevents intimal hyperplasia in native vessels and provided an organization of the collagen fibers (Leal et al., 2021). Due to the few studies performed with chitosan and elastin, more research should be conducted to provide information on the effects of these materials in the design of vascular grafts. Another material used relatively frequently is silk fibroin, which is a natural, biodegradable, and biocompatible polymer (Lovett et al., 2010; Aytemiz et al., 2013; Alessandrino et al., 2019; Jin et al., 2019b). This natural proteic material is not immunogenic in humans, favors angiogenesis (Alessandrino et al., 2019), and presents good cell compatibility and hydrophilicity responses (Liu et al., 2019; Jin et al., 2019b). Also, native blood vessels can be decellularized to obtain ECMs (Assmann et al., 2012; Dahan et al., 2017; Bai et al., 2019). These matrices maintain the biological properties of native blood vessels (Simon et al., 2003).

Based on the studies analyzed in this systematic review, it appears that cellular recruitment or endothelialization tended to be used in grafts with biological materials, but mechanical properties were superior for biodegradable polymers. The porosity of biodegradable polymers allows a customized graft design, as well as withstanding different pressures, twisting and stretching. However, they do not seem suitable for cell adhesion and proliferation. On the other hand, natural materials have the capacity for cell adhesion, but have poor mechanical properties. Nevertheless, both endothelialization and mechanical properties are essential for the long-term patency of TEVGs (Wu et al., 2018). For these reasons, a hybrid graft emerged. This graft is a type of TEVG formed by mixing biodegradable polymers and biological materials. An example of this type of hybrid graft was used by Hu et al. (2017) who designed grafts with PLCL, collagen, and elastin. Better results were obtained by the different authors who designed a hybrid graft. The hybrid strategy could be used to create a highly favorable composite material for TEVGs, since these grafts present the advantages of both materials.

### 4.4 Manufacturing Technology

There are different techniques to produce TEVGs, with electrospinning being the most commonly used method. This technique produces porous and fibrous scaffolds from polymers (Dvir et al., 2010), thereby enhancing the transfer of nutrients and residues through the scaffold. The alignment of the nanofibers allows the scaffold’s strength to be increased to promote cell alignment (Woods and Flanagan, 2014). The second most commonly used method is decellularization. Native blood vessels can be decellularized to obtain ECMs. These matrices maintain the biological properties of native blood vessels. Thus, the grafts contain functional proteins capable of promoting cell recruitment. However, the decellularization process can also damage the ECM (Simon et al., 2003), thus negatively affecting the integrity, and mechanical properties of the graft. On the other hand, lyophilization is a physical technique that reduces calcification, and provides a stable graft (Conconi et al., 2014; Bai et al., 2019). Nevertheless, mechanical properties, such as suture strength, are not sufficient for clinical use (Reinhardt et al., 2019). Another technology, known as “biotubes,” involves the development of blood vessels by subcutaneous implantation, and is associated with a biological defense mechanism (Rothuizen et al., 2016; Tseng et al., 2017; Furukoshi et al., 2019; Yamanami et al., 2019). Finally, some authors used the innovative 3D bioprinting technique to manufacture TEVGs (Itoh et al., 2015; Itoh et al., 2019; Jang et al., 2020). This technology provides an adequate cell distribution with a high cell density (Leal et al., 2021). However, results showed that 3D bioprinting designs TEVGs with a low mechanical property (Leal et al., 2021). Further, a scaffold may or may not be present in a graft (Campbell and Campbell, 2007), but it would have to be degradable to allow the growth of new tissue from the body itself (Chlupác et al., 2009). There are researchers who designed scaffold-free grafts, wherein tubular tissues were created with 3D bioprinting (Itoh et al., 2015; Itoh et al., 2019).

It should be noted that some studies have designed decellularized vascular grafts with ECs (Koennerker et al., 2012; McIlhenny et al., 2015; Pellegata et al., 2015). This combination avoids the drawbacks of this methodology, obtaining native-like ECM. Nevertheless, research presents electrospinning as the technology most widely used by the authors in the development of TEVGs. The porosity of the scaffold is essential to design a graft in which good cell adhesion and tissue regeneration take place. It is possible to create vascular grafts with conditions very similar to those of a native vessel due to the great advance of this technology. Electrospinning allows controlling the porosity of the scaffold, providing an excellent cell adhesion and proliferation. However, 3D bioprinting seems to be a promising technology because any 3D organ can be manufactured using a computer software that simulates the structure itself, in addition to selecting the material to be used for printing. Although further studies are needed to ensure the effectiveness of this method.

### 4.5 Cell Seeding and Cell Types

Cell seeding facilitates cell fixation and infiltration, thus improving graft endothelialization. There are different ways to perform cell seeding, such as static, dynamic, electrostatic, and magnetic techniques. Static or gravitational cell seeding involves direct application of the cell suspension into the scaffold (Ong et al., 2017b; Radke et al., 2018). However, this technique has a series of disadvantages, such as risk of contamination, non-uniform seeding, or the risk of platelet adhesion due to the use of adhesion molecules in the lumen, among others (Radke et al., 2018). The most commonly cell seeding technique was dynamic cell seeding, which enhances seeding efficiency, uniformity and scaffold penetration due to the use of rotational seeding, vacuum seeding, and fluid sheer stress (Ong et al., 2017b; Radke et al., 2018). However, adequate graft porosity is required for optimal utilization of dynamic cell seeding, especially with the use of vacuum pressure that draw cells in through the micropores of the TEVG (Ong et al., 2017b; Radke et al., 2018). The material properties of the TEVG are an important factor for electrostatic cell seeding, which uses a temporary positive charge on the negatively charged graft lumen (Ong et al., 2017b). Finally, magnetic cell seeding use magnetic beads and application of an external magnetic field. This novel technique improves the efficiency of cell seeding due to a better regulation of cellular distribution, in addition to providing faster cell culture (Radke et al., 2018). Nevertheless, beads may cause side effect, so they need to be analyzed to ensure the validity of this technique (Radke et al., 2018). These last two techniques of cell seeding need to be further evaluated to ensure long-term cell retention and other side effects after *in vivo* implantation, in addition to the fact that there are hardly any studies using these cell seedings.

In terms of cell type, mesenchymal stem cells (MSCs) can be obtained from different origins, such as adipose tissue, bone marrow, and umbilical vein blood (Radke et al., 2018). MSCs are a promising cell type for TEVG because improve the patency due to their anti-thrombogenic property, and they can also recruit ECs on site (Hashi et al., 2007; Zhao et al., 2012; Itoh et al., 2015). A wide variety of vascular cells could be obtained with induced pluripotent stem cells (iPSCs) because they could be induced into specific lineages (Radke et al., 2018; Luo et al., 2020), such as SMCs or ECs. Finally, BM-MNCs were the second most commonly used cell type by some of the authors (Hibino et al., 2011; Lee et al., 2014; Stacy et al., 2014; Pepper et al., 2017; Sugiura et al., 2018). BM-MNCs include ECs, MSCs, immune-related cells and hematopoietic stem cells, which main property is their anti-thrombotic effect (Radke et al., 2018). Although various cell types can be used, ECs are the most commonly used cell in the design of TEVGs (Cummings et al., 2012; Koennerker et al., 2012; McIlhenny et al., 2015; Pellegata et al., 2015; Ma et al., 2017), as they provide anticoagulant effects (Ju et al., 2017) and improve endothelialization (Radke et al., 2018). In the study by Ju et al. (2017), the authors compared the effects of EC on a vascular scaffold fabricated by electrospinning of polycaprolactone and type I collagen. The results showed that the control group (scaffold without EC) had no patency owing to the appearance of thrombosis, whereas in the experimental group (scaffold with EC), the graft patency was maintained during the 6-months study.

Nevertheless, cell seeding is currently a challenging field of research because the optimal number of cells for seeding is unknown. Therefore, there is controversy about the need and clinical relevance of cell seeding (Ong et al., 2017b). Furthermore, it has been determined that many of the seeded cells are lost, owing to either cell death or lack of union with other cells (Villalona et al., 2010). For this reason, many researchers have designed cell-free vascular grafts, scaffolds, or acellular vascular grafts. Based on the results, the development of tissue-engineered acellular grafts is a promising technique due to the wide variety of materials that can be employed, both natural materials and biodegradable polymers. These grafts have demonstrated good patency and regeneration potential, biocompatibility, and lack of immunogenicity (Smith et al., 2019). Additionally, TEVG scaffolds provide good mechanical strength and promote cellular proliferation and maturation (Ong et al., 2017b; Yeung et al., 2019). These scaffolds play a fundamental role in functional tissue regeneration because they interact with nearby biomolecule cells, regulating complete tissue regeneration. Also, the addition of vascular endothelial grow factors in the scaffold is very common in order to promote endothelization (Koobatian et al., 2016; Row et al., 2017; Smith et al., 2019).

In addition to all of the above, articles analyzed showed quite similar results in cell-based and cell-free TEVGs, in which the graft was effectively implanted and showed good mechanical and cell proliferation properties. However, cell-free TEVGs may correct some of the problems associated with the use of cells, such as immune rejection, long cell culture times, and avoidance of taking biopsies from patients. As mentioned, cell seeding is currently a challenging field of research, where insufficient cell seeding, damage or lack of cells can lead to the development of thrombosis and have poor patency rates. Cell-free scaffold is favorable for vascular graft design. In contrast to cell seeding, manufacturing techniques are evolving rapidly, and it is becoming easier and safer to design TEVGs.

Although a perfect material and manufacturing technology have not yet been identified, research in this field has become effective from a clinical point-of-view. The studies included in this review achieved to improve vascular grafts in a way that yielded very promising results compared with similar studies conducted previously.

### 4.6 Challenges of These Studies

Regarding the surgical outcomes, the researchers generally used small-diameter vascular grafts. However, it has been documented that 1) small-diameter TEVGs are especially prone to failure from thrombus adherence and vascular obstruction (Jin et al., 2019a; Jin et al., 2019b), and 2) current vascular grafts have a limited durability and patency (Iacobazzi et al., 2018). These circumstances seem to be related to poor endothelialization and smooth muscle layer remodeling (Jin et al., 2019b). Some of the strategies used to avoid thrombosis and subsequent graft stenosis and improve patency involve anticoagulant substances during surgery, such as heparin (Siemionow, 2015). However, some researchers did not use heparin during surgery. For this reason, we decided to analyze the relationship between the existence of thrombosis and the patency of the vascular grafts with respect to their diameter, as well as the use of heparin during surgery.

Several studies have been conducted to manufacture small TEVGs to achieve the phenotype of native blood vessels. Results in rodents showed that vascular grafts with larger diameters were patent, and grafts with smaller diameters were occluded. Contrary to expectations, rodents exhibited a greater onset of thrombosis in grafts with larger diameters. Among the analyzed studies, large-diameter vascular grafts had endothelial injury (Quint et al., 2012; Yang et al., 2016) or lack of EC (Assmann et al., 2012; Conconi et al., 2014; Gui et al., 2016; Luo et al., 2020). Therefore, any inappropriate movement may cause trauma to the graft and a blood flow mismatch. A complete endothelial layer plays a critical role in maintaining patency and provides anticoagulant effects (Ju et al., 2017; Wu et al., 2018).

In the porcine model, in contrast, it seems that there is a trend based on which vascular grafts with small diameters leads to thromboses. TEVG is a medical device in contact with the patient’s blood. Thus, the formation of thrombi constitutes a common type of failure. For this reason, it is important to use anticoagulants, antiplatelet agents, or both, to avoid the occurrences of thrombi (Jaffer et al., 2015; Siemionow, 2015).

As expected, the results also showed a correlation between the vascular graft patency and the absence of thrombi in mice, ovine, porcine, and canine models. Likewise, the lack of graft patency correlated with the occurrence of thrombi in the nonhuman (baboon) model. Therefore, considering all previous results, it appears that when a small-diameter TEVG is used, there is a tendency for the formation of thrombi and poor vascular graft patency.

Surgical experience and skills, differences in graft diameter from the native vessel, damage or lack of EC, tension in the suture line, damage during surgery, and hemodynamic factors leading to blood flow mismatch are (among others) potential risk factors that could lead to the appearance of thrombi and poor patency of vascular grafts (Pashneh-Tala et al., 2015), especially for small-diameter TEVGs. The surgery must be performed perfectly as any inappropriate movement can cause trauma to the graft. Additionally, a mismatch in the TEVG diameter could cause turbulence in the blood flow, thus triggering a coagulation cascade that can form thrombi.

Good graft porosity, good EC conditions, and the aforementioned recommendations are key factors associated with the prevention of the appearance of thrombosis and the achievement of good patency of vascular grafts.

The three studies on humans included in this review did not indicate the use of heparin. Specifically, 13/60 patients (21.67%) in the study by Kirkton et al. had to be treated for thromboses in the arteriovenous graft (Kirkton et al., 2019), and 1/25 patients (4%) in the study by Sugiura et al. had thrombi in the TEVG (Sugiura et al., 2018). These patients received anticoagulation therapy, such as aspirin or warfarin (Sugiura et al., 2018; Kirkton et al., 2019), which started 2 days after surgery and continued up to 3–6 months. A study by Hibino et al. (2010) on 25 human patients also employed the same anticoagulant therapy during implantation of TEVGs. The TEVG was composed of PGA and PLCL and was seeded with autologous BM-MNCs. The grafts measured between 12 and 24 mm and were implanted in the forms of extracardiac total cavopulmonary connection. After surgery, 24/25 patients (96%) had no evidence of thrombosis for 6 months, and 10/25 patients (40%) did not require long-term medication. This type of anticoagulation therapy has also been used extensively in animal model research. It should be noted that no correlation was found between the use of heparin and the onset of thrombi in the animal model studies investigated in this review. This fact may be attributed to the lack of information regarding the use of heparin during surgery in many of these studies, which made analysis difficult.

When PET or PTFE are used to design artificial vascular grafts, human patients require the long-term use of warfarin and aspirin (Giannico et al., 2006; Nakano et al., 2007; Kim et al., 2008). In contrast, one of the advantages of TEVGs is the fact that they allow antiplatelet or anticoagulation therapy to be skipped owing to the creation of autologous tissue (Hibino et al., 2010). However, the results showed that the use of these therapies to prevent thrombus formation remains relevant. Further, based on the animal models results, there is not yet a TEVG design that presents suitable mechanical and cellular recruitment conditions. Also, the few studies conducted on human studies showed a high percentage of thrombi. The use of TEVGs is not advisable in clinical studies due to tendency for thrombus formation and poor vascular graft patency.

### 4.7 Blood-Contacting Surfaces in TEVGs

In addition to all of the above factors, as well as the different materials used for TEVG design, there are other factors, such as the topography and architecture of the blood-contacting surface, that have a significant impact on thrombus formation. The topographic gradients of the material surface significantly modify the platelet adhesion and activation (Radke et al., 2018). Those surfaces with a rough topography cause that platelet are less adherent and activated with greater difficulty than smooth one (Hulander et al., 2013). Further, a surface with structured ridges and grooves affects platelet adhesion, which significantly reduces its thrombogenic effect (Radke et al., 2018). Others factors involved in the regulation of platelet adhesion, spreading, and activation are the mechanical properties of the surface that comes into contact with the blood. Stiffer surfaces are more prone to platelet adhesion and spreading (Radke et al., 2018). All of these factors should be taken into account to improve the design of TEVGs. However, articles studied in this systematic review did not include this information in their research, so it was not possible to analyze these data.

Moreover, authors had employed different strategies to produce successful blood-contacting surfaces in TEVGs. One of these strategies is to incorporate anti-thrombotic cells (ECs, MSCs, or BM-MNCs) and use safe cell seeding (dynamic cell seeding), which have a vital role in avoiding thrombus formation, as previously mentioned. Another strategy was the use of autologous cells, which avoid immune rejection. However, this strategy has a number of disadvantages, such as the need to perform a biopsy, long-term culture period, cells depend on the patient’s health, and age, among others (Radke et al., 2018). Finally, the use of heparin was used as strategy to successfully produce blood-contacting surfaces in TEVGs. As already indicated, this anticoagulation molecule regulates platelet activity and enhances biocompatibility (Aslani et al., 2020).

### 4.8 Futures Perspectives

The field of TEVG research is very extensive, with a wide variety of materials and techniques used in the development of these grafts. TEVGs manufacturing technology advances at an exponential pace, and it is expected that in a few years it will be possible to create vascular grafts with the same characteristics as native ones. In the future, more advanced biomaterial fabrication methods will be developed. These advances will allow the development of scaffolds with complex architecture and topography that could imitate the native ECM of the vessel and provide suitable mechanical properties tailored to clinical needs. In addition, the development of hybrid grafts now largely emulates the native blood vessel due to the combination of natural materials and biodegradable polymers, so the safe clinical use of TEVGs in human patients is getting closer.

Each TEVG analyzed in this systematic review has its own design. However, there are still many aspects that should be taken into account in the future. One of them is that there are a wide variety of animal models, so future research should be oriented according to the objective of the study. As mentioned above, each animal model has a number of limitations. Therefore, it is important to know the different characteristics of each animal model to be able to correctly interpret their results and their possible effects on human patients. In addition, most studies have been performed in young, healthy animals. Nonetheless, the clinical use of these grafts is usually carried out in elderly humans, and these patients often suffer from some type of disease, either renal (TEVGs for hemodialysis) or cardiovascular, in addition to concomitants disorders. Consequently, the design of TEVGs tested in a young and healthy animal model may behave differently in these patients. For this reason, future studies with experimental models with different pathologies should be included.

Substantial variability in TEVG implantation time has been observed, ranging from less than 1 h (Li et al., 2017) to 3 years (Kajbafzadeh et al., 2019). Most articles, however, have follow-up durations in the range between 1 and 6 months. This temporal analysis is very important because in human studies asymptomatic complications were diagnosed. Therefore, long-term research is needed to study all the side effects that these TEVGs could produce, thus achieving subsequent safe and satisfactory use. Additionally, patients generally require the use of vascular grafts immediately. For this reason, studies should be aimed at creating vascular grafts in a rapid and effective way.

TEVGs emerged as alternative replacement vessels for clinical applications. Small-diameter vascular grafts hold promise to overcome the limitations of synthetic grafts. As observed in this review, studies were mainly focused on the development of small-caliber TEVGs, but there is a tendency for thrombus formation and poor vascular graft patency. However, advances in TEVG fabrication technology may address these problems in the near future. Electrospinning showed very encouraging results, developing grafts with good patency and mechanical properties very similar or superior to native vessels. Moreover, it is now possible to 3D print a vascular graft with very satisfactory properties. Although these technologies are promising, the correct use of materials remains a challenge, and mechanical properties and cell recruitment of TEVGs need to be controlled in order to maintain long-term graft patency. All these issues should be further addressed to develop safe TEVGs for clinical applications. Therefore, future studies should be aimed at the development of new materials or combinations of them, as well as testing emerging technologies, and study the cell-material interaction to develop a suitable TEVG.

Finally, it is fundamental that the research should be aimed at designing TEVGs with similar or equal characteristics to the site to be replaced. For this purpose, images of the native vessel should be taken by MRI, computed tomography or any other medical imaging method that allows the dimensions and characteristics of the vessel to be replaced to be analyzed.

### 4.9 Limitations of the Study

This study has a number of limitations. Research on TEVG has grown exponentially, and a wide variety of results has been obtained. Therefore, because of the high multiplicity in the materials and technologies used in the development of these grafts, considering all studies is challenging. On the other hand, only three studies in human patients were included in the review, so it has not been possible to extract significant results. There is heterogeneity in the protocols and a lack of information in the results obtained from human patients and animal models, which hinders the analysis and interpretation of the data. Finally, the topography, architecture and mechanical properties of the blood-contacting surface could not be analyzed due to lack of data.

### 4.10 Conclusion

Although there is no ideal animal model for TEVG research, rodent models are the most commonly used animal model. Continuous progress is currently being made in the development of TEVGs, and hybrid grafting, and electrospinning are presented as the most promising technologies. Nevertheless, the major complication arising from the use of TEVGs is the occurrence of thrombosis and the lack of patency, which limits the implementation of TEVGs in clinical applications. These complications seem to be aggravated by the use of small-diameter TEVGs and the lack of anticoagulation therapy. *In vivo* studies are oriented toward the safety of these devices, pursuing a suitable design, and further clinical applications.

## Data Availability

The original contributions presented in the study are included in the article/Supplementary Material, further inquiries can be directed to the corresponding author.
