# Supplementary material for: Systematic Review of Tissue-Engineered Vascular Grafts
Source: Front Bioeng Biotechnol. 2021 Nov 3;9:771400. doi: 10.3389/fbioe.2021.771400 (PMC8595218; doi:10.3389/fbioe.2021.771400)
Supplement: Supplementary file 1 [file Table1.DOCX]

Supplementary Material

# Supplementary Data

***Full search strategy***

The search string used in this systematic review was:

PubMed:

*(("tissue engineer*") OR (tissue scaffold*) OR bioprint* OR print* OR (three-dimensional print*) OR (3D print*) OR (3-D print*) OR (three-dimension* print*) OR (3-dimension* print*)) AND (cardiovasc* OR (circulatory system)) AND Surg* AND (graft* OR prost* OR bypass) AND ((blood vessel) OR arter* OR vein OR vascular) AND ((animal model) OR human)*

Web of Science:

*TS=((("tissue engineer*") OR (tissue scaffold*) OR bioprint* OR print* OR (three-dimensional print*) OR (3D print*) OR (3-D print*) OR (three-dimension* print*) OR (3-dimension* print*)) AND (cardiovasc* OR (circulatory system)) AND Surg* AND (graft* OR prost* OR bypass) AND ((blood vessel) OR arter* OR vein OR vascular) AND ((animal model) OR human))*

# Supplementary Figures and Tables

## Supplementary Tables

**Table 1.** Eligibility criteria applied to the identified articles.

| **Exclusion Criteria** | **Inclusion Criteria** |
| --- | --- |
| Reviews | Results of the last 10 years |
| Articles written in a language different from English | Articles written in English |
| Expert opinions | Tissue-engineered vascular grafts |
| Letters | Studies using experimental animal models and human patients |
| Conference proceedings | Studies including surgical procedures and follow-up |
| Book chapters | Grafts implemented on a vascular vessel |
| *In vitro* studies | Grafts made of biodegradable polymers and natural materials |
| *Ex vivo* studies |  |
| Non-degradable grafts (e.g., polyethylene terephthalate (PET), Polytetrafluoroethylene (PTFE), expanded PTFE (ePTFE) grafts) |  |
| No inter-positioned grafts (e.g., vascular stents, patches, subcutaneous implantation) |  |
